# Commencement of New York College of Veterinary Surgeons

**Published:** 1886-04

**Authors:** 


### COMMENCEMENT OF NEW YORK COLLEGE OF VETERINARY SURGEONS.

The commencement exercises of the New York College of Veterinary Surgeons were held at the college building, 332 East 27th street, on Thursday evening, March 18, 1886.

Diplomas were granted to the graduating class as follows: George W. Gilbert, Long Island, John S. Shiffert, Pennsylvania, John J. Foy, New York City, J. T. D. Donnelly, Long Island, Otto A. A. von Lang, Brooklyn, N. Y., Fremont L. Russel, Maine, J. A. Breakell, M.D., New York City.

The Faculty Prize, a gold medal, was awarded to Geo. W. Gilbert for the best general examination. Dr. R. McLean's prize, a silver medal, was awarded to James McKee for general best junior examination Prof. Comstock's prize, a veterinary pocket case of instruments, was awarded to Geo. W. Gilbert for the best examination in anatomy. The Jenkins Prize, $10 worth of veterinary books, was awarded to J. T. D. Donnelly.

Prof. J. McLean, of the Faculty, addressed the students in his usual happy manner, and gave them some good advice. G. W. Gilbert delivered the valedictory.

The Spring Term will commence on March 29th, and continue until the middle of May.

Dr. Peters, having removed from the city, no longer holds any official connection with the college.

